# Bat Predation by Spiders

**DOI:** 10.1371/journal.pone.0058120

**Published:** 2013-03-13

**Authors:** Martin Nyffeler, Mirjam Knörnschild

**Affiliations:** 1 Section of Conservation Biology (NLU), Department of Environmental Sciences, University of Basel, Basel, Switzerland; 2 Institute of Experimental Ecology, University of Ulm, Ulm, Germany; Aarhus University, Denmark

## Abstract

In this paper more than 50 incidences of bats being captured by spiders are reviewed. Bat-catching spiders have been reported from virtually every continent with the exception of Antarctica (∼90% of the incidences occurring in the warmer areas of the globe between latitude 30° N and 30° S). Most reports refer to the Neotropics (42% of observed incidences), Asia (28.8%), and Australia-Papua New Guinea (13.5%). Bat-catching spiders belong to the mygalomorph family Theraphosidae and the araneomorph families Nephilidae, Araneidae, and Sparassidae. In addition to this, an attack attempt by a large araneomorph hunting spider of the family Pisauridae on an immature bat was witnessed. Eighty-eight percent of the reported incidences of bat catches were attributable to web-building spiders and 12% to hunting spiders. Large tropical orb-weavers of the genera *Nephila* and *Eriophora* in particular have been observed catching bats in their huge, strong orb-webs (of up to 1.5 m diameter). The majority of identifiable captured bats were small aerial insectivorous bats, belonging to the families Vespertilionidae (64%) and Emballonuridae (22%) and usually being among the most common bat species in their respective geographic area. While in some instances bats entangled in spider webs may have died of exhaustion, starvation, dehydration, and/or hyperthermia (i.e., non-predation death), there were numerous other instances where spiders were seen actively attacking, killing, and eating the captured bats (i.e., predation). This evidence suggests that spider predation on flying vertebrates is more widespread than previously assumed.

## Introduction

Bats have few natural enemies [1]. The most prominent bat enemies mentioned in the scientific literature are owls, hawks, and snakes [1]–[3]. Predation by a few large arthropods is occasionally documented in the literature as well [3]–[5]. In a cave in Venezuela, giant centipedes (Scolopendridae) have been observed killing and eating mormoopid and phyllostomid bats [5]. Whip spiders (Phrynidae) were observed feeding on dead phyllostomid bats in caves of the Caribbean, but it is not known whether this were cases of predation or scavenging ([6]; B. Fenton, pers. comm.). Despite their name, whip spiders do not belong to the order “spiders” (Araneae); instead they belong to the Amblypygi which is a separate arachnid order. Furthermore, cockroaches have been seen feeding on bat pups which have fallen to the floor [7]–[8]. In several technical books on chiropterology, accidental deaths of bats in spider webs have been reported [3], [9]–[12]. The observation of bat-catching by spiders is not that peculiar if we consider the fact that a number of larger-sized spiders are known to supplement their arthropod diet by occasionally preying on vertebrates. Fishing spiders (Pisauridae) have been reported capturing and devouring fish and frogs [13]–[14]. Some species of wolf spiders (Lycosidae), huntsman spiders (Sparassidae), tarantulas (Theraphosidae) and other mygalomorph spiders were observed killing and eating frogs and lizards [13], [15]–[19]. Predation on snakes and mice by tarantulas and comb-footed spiders (Theridiidae) has been mentioned in the literature [13], [20]. Furthermore, there are numerous reports of birds being killed in the large orb-webs of araneid and nephilid spiders, whereby the birds were either eaten by the spiders or not [21]–[29].

Deaths of bats in spider webs have been considered to occur very rarely. In two more recent papers, a web-building spider, *Argiope savignyi*, and a theraphosid spider, *Poecilotheria rufilata*, were each reported to predate on a small bat [30]–[31]. These authors hypothesized that bat captures and kills due to spiders might be more frequent than previously thought. To test this hypothesis, an extensive global literature survey on bat-catching spiders was conducted, along with an attempt to use web-based sources as well. The insights from this research are reviewed here.

## Methods

An extensive bibliographic search was conducted in order to find any information available on bat-catching spiders. The search was based largely on the Thomson-Reuters data base (Web of Science), Google Scholar, Google Books, ProQuest Dissertations & Theses, and Flickr image-hosting website (hosting more than 6 billion images). In addition to this, an internet search for blogger information on this topic was conducted. Bloggers who had posted photographs and reports on bat-catching spiders on the internet were contacted to get detailed information on their observations. Furthermore, the staff of bat hospitals was contacted to get information on bats rescued from spider webs. Finally, an inquiry among fellow arachnologists and chiropterologists was carried out to get access to unpublished reports on this topic. Many of these experts had conducted field studies for decades, and their feedback provided valuable information needed to assess how frequent incidences of bat catches by spiders might be. Altogether, 52 reports on bat-catching spiders could be gathered (Table 1). Twenty-three of these reports have previously been published in scientific journals, books, and a doctoral thesis.

**Table 1:** Fifty-two reports of bat-catching spiders based on literature and unpublished data (for more details see File S1).

Mortality agent (spider taxon)		Victim (bat taxon)		Observed spider/bat-interaction	Country	Source	report #
Species	Family	Species	Family				
*Avicularia urticans*	Theraphosidae	*Saccopteryx bilineata*	Emballonuridae	Bat captured, killed and eaten by spider	Peru	Rick West, pers. website	1
*Avicularia* sp.	Theraphosidae	*Myotis nigricans*	Vespertilionidae	Bat captured, killed and eaten by spider	Ecuador	George Schmid, flickr website	2
*Lasiodora parahybana*	Theraphosidae	Unidentified	Unidentified	Bat captured, killed and eaten by spider	Brazil	Rick West, pers. website	3
*Poecilotheria rufilata*	Theraphosidae	*Pipistrellus ceylonicus*	Vespertilionidae	Bat captured, killed and eaten by spider	India	[31]	4
*Heteropoda venatoria*	Sparassidae	*Pipistrellus* sp.	Vespertilionidae	Bat captured and killed by spider, but not eaten	India	[36]	5
*Dolomedes triton*	Pisauridae	*Myotis septentrionalis*	Vespertilionidae	Bat attacked by spider but able to escape after the spider was disturbed by photo-graphing researchers	USA	Phil Clem & Virgil Brack, pers. comm.	6
*Nephila clavipes*	Nephilidae	*Myotis* sp.	Vespertilionidae	Bat died in spider web	Costa Rica	Harald & Gisela Unger, pers. comm.	17
*Nephila clavipes*	Nephilidae	*Rhynchonycteris naso*	Emballonuridae	Several bats died in spider webs	Peru	Marjorie Weber, pers. comm.	7
*Nephila clavipes*	Nephilidae	*Rhynchonycteris naso*	Emballonuridae	Bat captured in spider web but freed by researcher	Costa Rica	Martina Nagy, pers. comm.	16
*Nephila clavipes*	Nephilidae	*Glossophaga* sp.	Phyllostomidae	Bat captured in spider web	Costa Rica	getty images film 2010	20
*Nephila clavipes*	Nephilidae	*Saccopteryx bilineata*	Emballonuridae	Bat captured in spider web but freed by researcher	Panama	Maria Eckenweber, pers. comm.	21
*Nephila clavipes*	Nephilidae	Unidentified	Unidentified	Bat died in spider web	Colombia (site 1)	Dario Hernando Gutierrez, pers. comm.	8
*Nephila clavipes*	Nephilidae	Unidentified	Unidentified	Bat died in spider web	Colombia (site 2)	Dario Hernando Gutierrez, pers. comm.	9
*Nephila clavipes*	Nephilidae	Unidentified	Unidentified	Bat died in spider web	Guatemala	Sam Bloomquist, pers. comm.	11
*Nephila clavipes*	Nephilidae	Unidentified	Unidentified	Bat captured in spider web but able to escape prior to being bitten by spider	Costa Rica	Marco Mallo & Carmen Díez, pers. comm.	19
*Nephila pilipes*	Nephilidae	*Pipistrellus abramus*	Vespertilionidae	Bat captured in spider web, killed and eaten by spider	Hong Kong	[40]	29
*Nephila pilipes*	Nephilidae	*Pipistrellus abramus*	Vespertilionidae	Several bats died in spider webs	Hong Kong	Gary Ades, pers. comm.	30
*Nephila pilipes*	Nephilidae	*Hipposideros pomona*	Hipposideridae	Bat died in spider web	Hong Kong	Gary Ades, pers. comm.	31
*Nephila pilipes*	Nephilidae	*Rhinolophus cornutus orii*	Rhinolophidae	Bat captured in spider web, killed and eaten by spider	Japan	*Asahi* Newspaper, 19 September 2007	35
*Nephila pilipes*	Nephilidae	*Hipposideros ater* ?	Hipposideridae	Bat captured in spider web, killed and eaten by spider	Australia	Carmen Fabro, pers. comm.	39
*Nephila pilipes*	Nephilidae	*Pipistrellus* sp.	Vespertilionidae	Bat captured in spider web and bitten by spider; it survived after being freed by researchers	Papua New Guinea	[11]	45
*Nephila pilipes*	Nephilidae	Unidentified	Unidentified	Bat captured in spider web, killed and eaten by spider	China	[39]	28
*Nephila pilipes*	Nephilidae	Unidentified	Vespertilionidae	Bat captured in spider web, killed and eaten by spider	Hong Kong	Carol S.K. Liu, pers. comm.	32
*Nephila pilipes*	Nephilidae	Unidentified	Unidentified	Bat died in spider web	Hong Kong	Anonymous blogger	33
*Nephila pilipes*	Nephilidae	Unidentified	Unidentified	Bat captured in spider web, killed and eaten by spider	Vietnam	Padraig Larkin, blog 2006	36
*Nephila pilipes*	Nephilidae	Unidentified	Unidentified	Bat captured in spider web, killed and eaten by spider	Sri Lanka	[104]	38
*Nephila pilipes*	Nephilidae	Unidentified	Unidentified	Bat captured in spider web and bitten by spider; it died after being freed by researchers	Australia	[124]–[125]	40
*Nephila* sp.	Nephilidae	*Nyctophilus gouldi*	Vespertilionidae	Bat died in spider web	Australia	[126]	43
*Nephila* sp.	Nephilidae	*Hipposideros ater*	Hipposideridae	Bat died in spider web	Australia	[105]	44
*Nephilengys cruentata*	Nephilidae	Unidentified	Unidentified	Bat captured in spider web but able to escape prior to being bitten by spider	Swaziland	Donald Schultz, pers. comm.	47
*Araneus bilineatus*	Araneidae	*Pipistrellus abramus*	Vespertilionidae	Several bats died in spider webs	China	[106]	26
*Araneus heraldicus*	Araneidae	*Pipistrellus abramus*	Vespertilionidae	Several bats died in spider webs	China	[106]	27
*Argiope savignyi*	Araneidae	*Rhynchonycteris naso*	Emballonuridae	Bat captured in spider web, killed and eaten by spider	Costa Rica	[30]	14
*Argiope savignyi*	Araneidae	*Rhynchonycteris naso*	Emballonuridae	Bat died in spider web	Costa Rica	Mirjam Knörnschild, unpubl. data.	15
*Eriophora fuliginea*	Araneidae	*Myotis nigricans*	Vespertilionidae	Bat captured in spider web, killed and eaten by spider	Panama	[101]	22
*Eriophora fuliginea*	Araneidae	*Myotis nigricans*	Vespertilionidae	Bat captured in spider web, killed and eaten by spider	Panama	[8]	23
*Eriophora fuliginea*	Araneidae	*Myotis nigricans*	Vespertilionidae	Bat captured in spider web, killed and eaten by spider	Panama	[37]	24
*Eriophora transmarina*	Araneidae	Unidentified	Unidentified	Several bats died in spider webs	Australia	[125]	42
*Eriophora* sp. ?	Araneidae	*Centronycteris centralis*	Emballonuridae	Bat captured in spider web, killed and eaten by spider	Belize	Carol Farneti-Foster, pers. comm.	12
*Eriophora* sp. ?	Araneidae	Unidentified	Unidentified	Bat captured in spider web and wrapped by spider	Belize	National Geographic film 1998	13
*Eriophora* sp. ?	Araneidae	Unidentified	Unidentified	Bat captured in spider web and wrapped by spider	Central America	Nature (PBS) film 1985	25
*Eriophora* sp. ?	Araneidae	Unidentified	Unidentified	Bat captured in spider web, killed and eaten by spider	Costa Rica	Cassidy Metcalf, pers. comm.	18
*Parawixia dehaani*	Araneidae	Unidentified	Unidentified	Bat captured in spider web, killed and eaten by spider	Hong Kong	Gary Ades, pers. comm.	34
Unidentified	Araneidae	*Pipistrellus* sp.	Vespertilionidae	Bat died in spider web	Germany	German tabloid *BILD*, May 2011	51
Unidentified	Web-builder	*Cyttarops alecto*	Emballonuridae	Bat captured in spider web	Columbia	[127]	10
Unidentified	Web-builder	*Tylonycteris pachypus*	Vespertilionidae	Bat captured in spider web	Malaysia	[128]	37
Unidentified	Web-builder	*Chalinolobus gouldii* ?	Vespertilionidae	Bat died in spider web	Australia	[125]	41
Unidentified	Web-builder	*Nycticeinops schlieffeni*	Vespertilionidae	Bat captured in spider web but freed by researcher	Malawi	[42]	46
Unidentified	Web-builder	*Neoromicia nana*	Vespertilionidae	Bat died in spider web	Sierra Leone	[41]	48
Unidentified	Web-builder	*Pipistrellus hesperus*	Vespertilionidae	Bat died in spider web	USA	[44]	49
Unidentified	Web-builder	*Myotis austroriparius*	Vespertilionidae	Several bats died in spider webs	USA	[43]	50
Unidentified	Web-builder	*Pipistrellus pipistrellus*	Vespertilionidae	Bat captured in spider web; it died after being freed by researchers	England	Graham Street, pers. comm.	52

The species name *Vespertilio irretitus* has changed to *Pipistrellus abramus* (see [32]).

During these inquiries we got access to several previously unpublished photographs of bat-catching spiders. These photographs were shown to established bat and spider taxonomists for identification of the bats and spiders, respectively. In a few cases photographs of habitats were sent to vegetation specialists for proper habitat classification. Nomenclature follows [32]–[33]. Spiders reported in this paper are divided into two major groups based on foraging mode (*sensu* Gertsch [14]): “Hunting spiders” (i.e., spiders that forage without the use of a catching web) and “Web-building spiders” (i.e., spiders that forage using a catching web). Data on spider weight and size as well as bat weight, wingspan, foraging mode and echolocation call frequency were taken from the literature when available. Report numbers used in the results, tables and figures refer to the respective detailed report description (see File S1).

## Results

### Geographic Distribution of Bat Catches by Spiders

**Figure 1:**
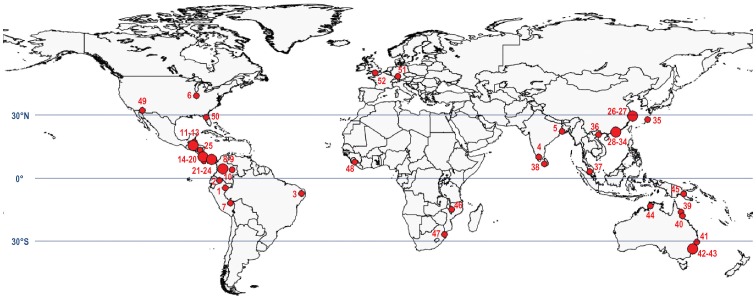
Geographic distribution of bat catching spiders worldwide. The map depicts the locations were spiders were observed catching bats (red dots). Large red dots indicate that several reports originated from the same geographic region. Numbers refer to the detailed report description (see File S1).

Incidences of bats being captured by spiders have been reported from virtually every continent with the exception of Antarctica (Table 1). Seventy-seven percent of the incidences listed in Table 1 were witnessed in regions of tropical climate between latitude 23° N and 23° S (∼90% of the cases occurred between latitude 30° N and 30° S; Fig. 1). The prevalence of such events in the warmer areas of the globe may be explained, among others, by the fact that the vast majority of spider species capable of catching bats are giant theraphosids, nephilids, and araneids who have their main area of distribution in the tropical and subtropical regions [20], [33]–[35].

**Figure 2:**
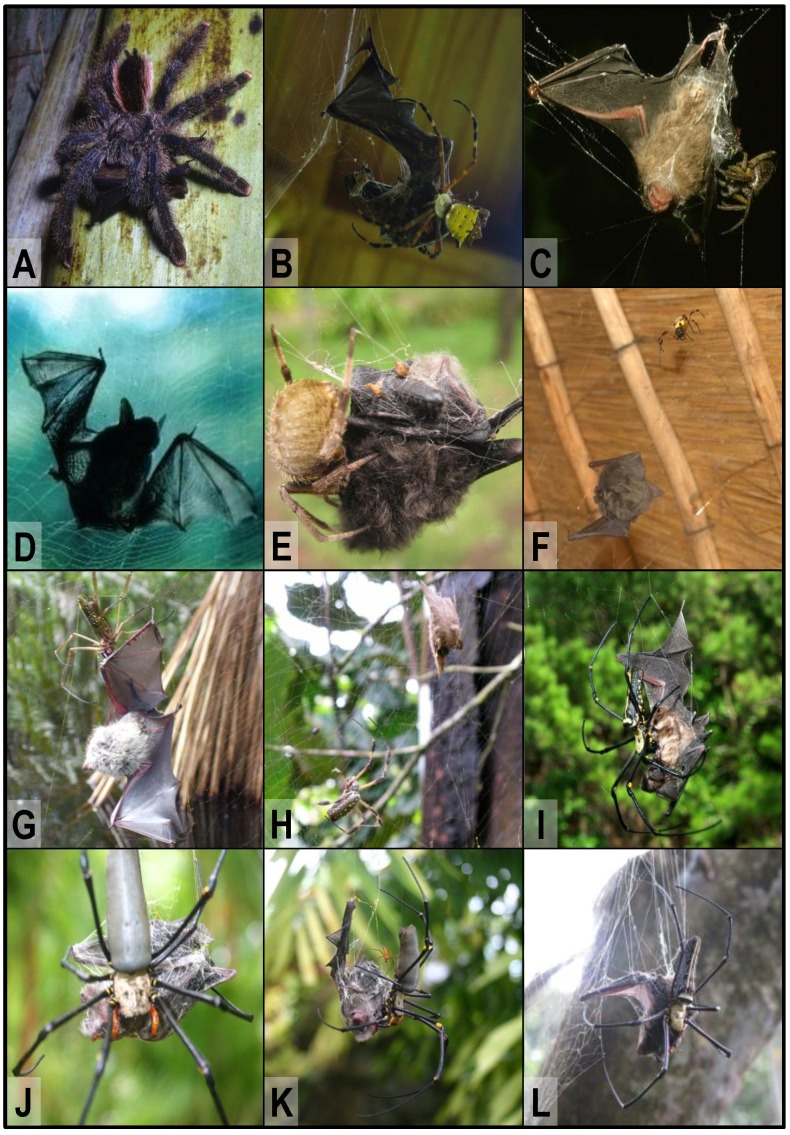
Bats caught by spiders. A - Adult female *Avicularia urticans* feeding on a Greater Sac-winged Bat (*Saccopteryx bilineata*) on the side of a palm tree near the Rio Yarapa, Peru (photo by Rick West, Victoria, Canada; report # 1). **B -** Adult Proboscis Bat (*Rhynchonycteris naso*) entangled in a web of *Argiope savignyi* at the La Selva Biological Station, northern Costa Rica (photo by Mirjam Knörnschild, Ulm, Germany; report # 14). **C -** Dead bat (presumably *Centronycteris centralis*) entangled in an orb-web in Belize (photo by Carol Farneti-Foster, Belice City, Belize; report # 12). **D -** Dead bat (*Myotis* sp.) entangled in a web of *Nephila clavipes* in La Sirena, Corcovado National Park, Costa Rica (photo by Harald & Gisela Unger, Köln, Germany; report # 17). **E -** A bat caught in the web of an araneid spider (possibly *Eriophora* sp.) in Tortuguero National Park, Costa Rica (photo by Cassidy Metcalf, USA; report # 18). **F -** Live bat trapped in web of *Nephilengys cruentata* in a thatch roof at Nisela Lodge, Swaziland (photo by Donald Schultz, Hollywood, USA; report # 47). **G -** Volant juvenile Proboscis Bat (*Rhynchonycteris naso*) entangled in web of *Nephila clavipes* photographed in a palm swamp forest near Madre de Dios, Peru (photo by Sam Barnard, Colorado Springs, USA; report # 7). **H -** Dead bat entangled in web of a female *Nephila clavipes* in tropical rainforest in the middle of the Rio Dulce River Canyon near Livingston, Guatemala (photo by Sam & Samantha Bloomquist, Indianapolis, USA; report # 11). **I -** Dead bat (*Rhinolophus cornutus orii*) caught in the web of a female *Nephila pilipes* on Amami-Oshima Island, Japan (photo by Yasunori Maezono, Kyoto University, Japan; report # 35). **J, K -** A small bat (superfamily Rhinolophoidea) entangled in web of *Nephila pilipes* at the top of the Cockatoo Hill near Cape Tribulation, Queensland, Australia (photo by Carmen Fabro, Cockatoo Hill, Australia; report # 39). The spider pressed its mouth against the dead, wrapped bat, indicating that it was feeding on it. A *Nephila pilipes* male also present in the web (**K**) may have been feeding on the bat as well. **L -** Dead vespertilionid bat entangled in the web of a female *Nephila pilipes* in the Aberdeen Country Park, Hong Kong (photo by Carol S.K. Liu from AFCD Hong Kong, China; report # 32).

There are six reports on bats being captured by hunting spiders. Tarantulas (Theraphosidae) of the genus *Avicularia* have been observed eating small bats in tropical rainforest areas in Peru (Fig. 2A; report # 1) and eastern Ecuador (report # 2), respectively ([18]; Rick West, pers. website; G. Schmid, flickr image hosting website). A large tarantula of the genus *Lasiodora* was observed eating a bat on the forest floor in northeastern Brazil (R. West, pers. comm.; report # 3). Moreover, a large Reddish Parachute tarantula, *Poecilotheria rufilata*, was reported to predate on a small bat in Kerala, India ([31]; report # 4). Furthermore, a huntsman spider *Heteropoda venatoria* (Sparassidae) was observed capturing and killing a small bat in a shed near Kolkata, India ([36]; report # 5). This spider had apparently not fed on the bat which may be explained by the fact that the observer interfered by capturing spider and bat and placing them into a glass jar (see File S1). An attempt by a large fishing spider *Dolomedes triton* (Pisauridae) to kill a bat pup has been witnessed below a bridge in Indiana, USA (P. Clem & V. Brack, pers. comm.; report # 6). However, in this latter case the predation attempt failed probably because the spider was frightened by the presence of the photographing observers (see File S1). All other reports refer to web-building spiders.

There are 19 reports of Neotropical bats being captured in orb-webs of large araneids or nephilinids. These reports refer to incidences witnessed in Peru (report # 7), Colombia (report # 8–10), Guatemala (report # 11), Belize (report # 12–13), Costa Rica (report # 14–20), Panama (report # 21–24), or elsewhere in Central America (report # 25) and are depicted in Fig. 2B–E, G and H. Several of these observations were made by researchers stationed at the Los Amigos Biological Station in Peru, the Biological Stations La Selva and La Sirena in Costa Rica and the Smithsonian Tropical Research Station Barro Colorado Island in Panama, which are all located in lowland tropical rainforest ([8], [30], [37]–[38]; M. Knörnschild, unpubl. data; M. Eckenweber, pers. comm.; M. Mallo & C. Díez, pers. comm.; M. Nagy, pers. comm.; H & G. Unger, pers. comm.; M. Weber, pers. comm.). In the incidences witnessed in Costa Rica and Panama, the spiders had constructed their webs on the outside of or in close proximity to buildings inhabited by bat colonies ([8]; M. Knörnschild, unpubl. data; C. Metcalf, pers. comm.).

A second geographic region where bat-catching by web-building spiders has frequently been reported (13 reports; 2I and L) is eastern and southeastern Asia including locations in China (report # 26–34), Japan (report # 35), Vietnam (report # 36), Malaysia (report # 37), and Sri Lanka (report # 38). Here, bat-catching by spiders have been witnessed particularly often in the eastern coastal area of China, specifically in parks and forests of the Greater Hong Kong area ([39]–[40]; G. Ades, pers. comm.; C.S.K. Liu, pers. comm.; Fig. 2L).

A third geographic region where bat-catching by web-building spiders was repeatedly witnessed (seven reports) is the area of Australia (report # 39–44) and Papua New Guinea (report # 45). Most Australian incidences were observed in the coastal areas of New South Wales and Queensland (Fig. 2J–K).

Three incidences of bat captures by web-building spiders have been reported from Africa ([41]–[42]; D. Schultz, pers. comm.; Fig. 2F; report # 46–48). Only two incidences of bat catches by web-building spiders witnessed in North America have been reported so far ([43]–[44]; report # 49–50), and these both refer to warm areas in the southern USA. Incidences of bat catches by orb-weaving spiders are unknown from the northern part of North America (B. Fenton, pers. comm.). Likewise, incidences of this type have not been reported in the Ukrainian and Russian scientific literature (A.T. Bashta, pers. comm.). Only two incidences of bats being captured in spider webs have been reported from Europe (report # 51–52). In one case, a dead bat was found entangled in the web of an orb-weaving spider on a building site near Stuttgart, Germany (German tabloid *BILD*, May 2011). Another incidence of a bat caught in a spider web was observed on the Isle of Wight, South East England (G. Street, pers. comm.).

### Which Spider Species are Involved in Bat Catches?

Bat-catching spiders belong to the araneomorph families Nephilidae (golden silk orb-weavers), Araneidae (orb-weaver spiders), Sparassidae (huntsman spiders), and the mygalomorph family Theraphosidae (tarantulas). Furthermore, an attack attempt by an araneomorph hunting spider of the family Pisauridae (fishing spiders) was witnessed. Seventy-three percent of the known incidences of bat catches were attributable to orb-weaving spiders, 15% to unidentified web-building spiders, and 12% to hunting spiders (Table 1).

The dominant group of bat-catching spiders are giant orb-weavers of the genus *Nephila* (Nephilidae). These spiders are forest-dwellers that reach a legspan of 10–15 cm and a weight of ∼1–7 g ([34], [45]; Table 2). They are diurnally and nocturnally hunting [46]–[47]; feeding was found to be most intense in the time between sundown and midnight [46]. *Nephila* spp. spin strong webs with a diameter of up to 1.5 m at a height of 1 to 6 m above the ground [34], [47]–[48]. On certain locations, where females aggregate, several webs are built connected to each other, which may result in a web area of many square meters [49]. Of the 15 valid species in the genus *Nephila* (see [34]) only two species - namely *Nephila clavipes* and *Nephila pilipes* - have been reported so far to be engaged in bat catching. It can be assumed, however, that other *Nephila* spp. catch bats as well. Up to the present, the Neotropical *Nephila clavipes* has not been proven to feed on the bats captured in its webs. This species is, on average, significantly smaller than the Asia-Pacific species *Nephila pilipes* [50]–[52]. One might speculate that the smaller-sized *N. clavipes* perhaps is less successful in subduing bats (H. Höfer, pers. comm.). It appears that the female *Nephila* spiders depicted in Fig. 2H, I, and L were each missing a leg. Leg loss in adult female *Nephila* spiders is a well-known phenomenon [53]. It cannot be ruled out that some of the leg losses occurred during aggressive encounters between spiders and bats trying to defend themselves after being entangled in spider webs.

**Table 2:** Fresh weight and body length (cephalothorax plus abdomen) of adult spiders reported to catch bats (arranged in alphabethical order).

Spider species	Weight [g]	Body length [cm]	Source	report #
*Araneus bilineatus*	unknown	unknown	-	26
*Araneus heraldicus*	unknown	unknwon	-	27
*Argiope savignyi*	∼0.5 *	1.3–1.8	[129]	14–15
*Avicularia* sp.	10–30	5–7	[67]	1–2
*Dolomedes triton*	1	2	[130]	6
*Eriophora fuliginea*	1.4	3	[101]	22–24
*Eriophora transmarina*	unknown	2.5	www.findaspider.org.au/find/spiders/105.htm	42
*Heteropoda venatoria*	2–6.5	2–4	[63]	5
*Lasiodora parahybana*	up to >100	9–10	[68]	3
*Nephila clavipes*	1–3	2–3.5	[46]–[47]	7–9, 11, 16–17, 19–21
*Nephila pilipes*	2–7	4–5	[51], [53]	28–33, 35–36, 38–40, 45
*Nephilengys cruentata*	0.5–1.6	2.5	[60], [62]	47
*Parawixia dehaani*	unknown	2	[131]	34
*Poecilotheria rufilata*	28–85	6.5	[31]	4

*Epeira bilineata* and *Epeira heraldica* are now termed *Araneus bilineatus* and *Araneus heraldicus* and placed in the family Araneidae under *Nomina dubia* [33]. * Weight estimated using data for similar-sized adult female *Argiope argentata* [132].

A second dominant group of bat-catching spiders are large orb-weavers of the genus *Eriophora* (Araneidae) that spin strong vertical orb-webs of up to 1.5 m diameter [8], [37]–[38], [54]–[55]. These spiders, that reach a legspan of 5–8 cm and a weight of >1 g at favourable web sites (Table 2), are forest-dwellers like the *Nephila* spp. The *Eriophora* spp. are nocturnal spiders that hide in a leaf retreat during the daytime hours and start spinning their webs just after sunset [8], [37], [54]. They stay at the hub of the web all night long, the majority of their feeding activity taking place at the beginning of the night [56]. The genus *Eriophora* is represented in the Neotropics with four species [33]. A congener from Australia, *Eriophora transmarina*, has been witnessed catching bats as well (Table 1). Several araneids (genera *Eriophora* and *Caerostris*) spin orb-webs of extraordinary size and tensile strength suspended upon bridge lines of several meters, which led to the assumption that such web gigantism might have evolved as an adaptation for capturing flying vertebrates such as bats and birds [57]–[59]. In the case of *Caerostris darwini*, an araneid which spins giant orb-webs of up to 2 m diameter across rivers in Madagascar suspended upon bridge lines exceeding 20 meters in length, chiropterophagy could not be evidenced so far [58]–[59]. However, in this latter study the sample size of recorded predation events was rather small [58]–[59]. In the habitats of *Caerostris darwini*, several species of small riverine bats of the families Vespertilionidae and Emballonuridae occur who would be available as potential prey at least for a few weeks per year during which time their volant juveniles (weighing ∼2.5–4 g) are within the spiders’ prey size range (S. Goodman, pers. comm.; P.A. Racey, pers. comm.). Quantitative prey analyses of *Caerostris darwini* at various seasons (with a high enough sample size to not miss rare events) are urgently needed to answer the question whether this spider is preying exclusively on larger-sized flying insects (as suggested by [58]–[59]) or whether it supplements its diet by occasional catches of flying vertebrates.

*Nephilengys cruentata*, another bat-catching nephilid (Table 1), weighs 0.5–1.6 g (Table 2). It is a synanthrophic spider that spins orb webs (with up to 1 m diameter) on the corners of walls and ceilings of buildings [60]–[62]. *Nephilengys* might be less efficient in bat catching. The only reported incidence where a bat got entangled in a *Nephilengys* web ended with the bat’s successful escape. Nevertheless this spider species should not be ruled out as a potential predator of small bats; it is known to catch and eat various types of vertebrates including small birds [25]–[26], [62].

Other orb-weavers reported to have caught bats in their webs are found in the araneid genera *Argiope* and *Parawixia* (Table 1). These lighter spiders, in comparison to *Nephila* spp. and *Eriophora* spp., spin webs of much smaller size and may be engaged in bat catches less frequently, although it must be noted that *Argiope* spp. have repeatedly been observed killing (and sometimes eating) small birds in North America and elsewhere (e.g. [21], [29]).

*Heteropoda venatoria*, a sparassid spider reported capturing a pipistrelle in India, has an adult legspan of 7–12 cm and weighs between 2–6.5 g [63]–[64]. Other larger-sized cave-dwelling *Heteropoda* spp. (with a legspan of 20–30 cm), likewise suspected to be predators of bats, are expected to be even heavier though body mass data for these latter species are missing [65]–[66]. Based on their estimated size, the theraphosids *Avicularia* spp. engaged in bat-eating may have weighed between 10–30 g (compare [67] for body mass data of theraphosids), whereas *Lasiodora parahybana*, another bat-eating theraphosid, may have weighed even more [68]. The theraphosid *Poecilotheria rufilata* that was observed preying on a bat in India may have weighted between 28–85 g [31]. These giant sparassids and theraphosids are fully equipped with the toxins and enzymes needed to subdue and devour small vertebrate prey [14]–[18], [20]. The occasional capture of a bat should therefore come as no surprise, though these spiders are actually known to feed predominantly on arthropods [14], [64], [69]. Another group of hunting spiders that might be capable of attacking and killing small bats are a few large-sized species in the superfamily Lycosoidea (e.g., genera *Hogna* and *Dolomedes*) whose adult females weigh between 1–2 g [70]. Such huge hunters might occasionally attack neonatal bats fallen from the roost. An incident of this type, where a large pisaurid (*Dolomedes triton)* was attacking an immature bat, was witnessed in Indiana, USA (report # 6).

### Which Bat Species are Captured by Spiders?

So far, microbats from five families are known to have been captured by spiders in the field (Table 1): Vespertilionidae (plain-faced bats), Emballonuridae (sheath-tailed bats), Rhinolophidae (horseshoe bats), Hipposideridae (Old World leaf-nosed bats) and Phyllostomidae (New World leaf-nosed bats). In 31% of the reported incidences the captured bats remained unidentified. The majority of identifiable captured bats belonged to the families Vespertilionidae (64% of reports) or Emabllonuridae (22%), whereas only few reports existed for Hipposideridae (8%) and one for Rhinolophidae (3%). The capture of phyllostomid bats was only reported once (report # 20) or maybe twice (report # 11). In the latter, uncertain report, concerning a small brown-coloured bat entangled in a spider web in Guatemala (Fig. 2H; report # 11), the captured bat might have been a juvenile fruit-eating phyllostomid bat though the features needed for a positive identification were not sufficiently recognizable in the photo (A. Gardner, pers. comm.). The Old World flying foxes (Pteropodidae) have never been reported to get captured or killed by spiders except for one report from captivity. Liat [71] reported that neonates of the Lesser Dawn Bat, *Eonycteris spelaea* were offered to a theraphosid, *Coremiocnemis brachyramosa* (subfamily Selenocosmiinae), in captivity. The neonatal bats were eaten readily by this theraphosid. It is therefore possible that small or immature flying foxes are caught and eaten by spiders in the field as well. Due to the fact that the incidence reported by Liat [71] was not witnessed in the field, it was not included in Table 1.

The majority of bats captured by spiders represented species that are among the most common bats in their respective geographic region (e.g., *Pipistrellus* spp. in various parts of the world; *Myotis nigricans* and *Rhynchonycteris naso* in the Neotropics). Nevertheless, rare species may sometimes get caught in spider webs as well (e.g., *Centronycteris centralis*; compare [72]).

The majority of bats entangled in spider webs were small species with a wingspan of 10–24 cm and an adult weight of 3–8 g (Table 3). It is noteworthy that adult bat weight can vary by several grams depending on the bat’s feeding status and it is plausible that only the lightest individuals of any given bat species get entangled in spider webs; moreover, some of the captured bats were juveniles or subadults (reports # 7, 15–16, 20–23, and 50) and thus presumably much lighter than the weights for adults presented in Table 3. Some of the entangled bats are among the smallest bats on earth (e.g., *Tylonycteris pachypus* weighs only 3 g; [73]). Likewise, the neonatal *Myotis austroriparius* bats found entangled in spider webs in a study in Florida (see [43]) weighed only 3 g [74]. Large bats are missing in Table 3 because of these bats’ capability to fly right through a web (see below). Most of the species listed in Table 3 are known to roost in buildings, caves, and tree holes.

**Table 3:** Fresh weight and wingspan of bat species reported to be captured by spiders (arranged in alphabethical order).

Bat species	Weight (g)	Wingspan (cm)	Source	report #
*Centronycteris centralis*	6	∼24–27*	[133]	12
*Chalinolobus gouldii*	15	33.1	[86]	41
*Cyttarops alecto*	6	unknown	[127]	10
*Eonycteris spelaea* (neonatal)	8	N/A	[134]	captive evidence
*Glossophaga sp.*	11**	25.2**	[86]	20
*Hipposideros ater*	4–6	22.8	[135]–[136]	39, 44
*Hipposideros pomona*	5–8	26.0	[137]–[138]	31
*Myotis austroriparius* (neonatal)	3	N/A	[74]	50
*Myotis nigricans*	4	21.0	[86]	2, 22–24
*Myotis septentrionalis* (immature)	2	N/A	P. Clem & V. Brack, pers. comm.	6
*Neoromicia nana*	3–4	20.6	[86], [139]	48
*Nycticeinops schlieffeni*	5	22.4	[86]	46
*Nyctophilus gouldi*	11	31.1	[86]	43
*Pipistrellus abramus*	5–8	10.0	[140]	26–27, 29–30
*Pipistrellus ceylonicus*	8	25.6	[86]	4
*Pipistrellus hesperus*	4	19.0	[86]	49
*Pipistrellus pipistrellus*	5	21.8	[86]	52
*Rhinolophus cornutus*	4	18.0	[141]–[142]	35
*Rhynchonycteris naso*	4	23.9	[86]	7, 14–16
*Saccopteryx bilineata*	8	27.5	[86]	1, 21
*Tylonycteris pachypus*	3	19.0	[73]	37

The data refer to adults if not indicated otherwise. The species name *Vespertilio irretitus* has changed to *Pipistrellus abramus* (see [32]).

* Wingspan estimation based on data from *Rhynchonycteris naso* (smaller than *Centronycteris centralis*) and *Saccopteryx bilineata* (larger than *Centronycteris centralis*) published in [86].

** Reported values refer to data from *Glossophaga soricina*.

Eighty-five percent of the reports with identifiably bats entangled in spider webs refer to species classified as “aerial insectivorous bats” (Table 4). Such bats (e.g., *Pipistrellus* spp. and *Myotis nigricans*) feed aerially on flying insects; non-volant prey such as spiders is almost entirely missing in their diets [75]–[77]. In contrast, there are some bats that feed heavily on web-building spiders (e.g., *Phoniscus papuensis*, *Myotis emarginatus*, *Myotis nattereri*, *Myotis bechsteinii*, *Myotis keenii*, *Myotis lucifugus*, *Myotis aurascens*; see [78]–[85]). These are all bats with an average adult weight of 5–10 g and a wingspan of 21–27 cm [81], [86]. In particular, the Golden-tipped Bat (*Phoniscus papuensis* [formerly termed *Kerivoula papuensis*]) and Geoffroy’s Myotis (*Myotis emarginatus*) are considered to be true spider specialists with spiders making up>75% of their total prey [81], [85], [87]. These bats, which glean spiders from webs are characterized by high flight agility and manoeuvrability at low speed, using high frequency echolocation calls (>100 kHz start frequency) to detect spider prey and to avoid accidential crashes ([81]; [88]; G. Jones, pers. comm.). One might expect such highly specialized foragers to be sufficiently well adapted to avoid collisions with spider webs. However, studies in New South Wales (Australia) and Bavaria (Germany) revealed that gleaning insectivorous bats (i.e., *Phoniscus papuensis* and *Myotis emarginatus*) captured in harp traps and mist nets, had often spider web material attached to their body fur and wing membrane, indicating that these bats frequently strike spider webs while gleaning spiders suspended in webs or while accidentally encountering webs during their flights through the cluttered forest vegetation [87], [89]. In these latter studies, the web material must have originated from smaller-sized spider webs that were not strong enough to withstand the bats’ kinetic energy without breaking (“low energy absorbing webs” sensu Craig [90]). Analyses of faecal pellets likewise confirm that these bats prey on orb-weavers of rather small size (∼2–10 mm in length; [80], [91]), suggesting that they select smaller-sized webs for their gleaning attempts. Quite a number of studies on spider-eating *Myotis* bats were conducted in geographic areas such as Europe and northern parts of North America [78]–[79], [82]–[83], [85], [89], [91]–[94] where huge araneid and nephilid spiders do not occur (compare [33]), implying that in these areas gleaning bats presumably face little danger of being caught and killed by web-building spiders. But in some tropical areas of Australia, where *Phoniscus papuensis* does occur sympatrically with huge nephilid spiders, incidences of *Phoniscus papuensis* being accidentally ensnared and killed in *Nephila* webs would be imaginable, though nothing has been reported about this so far.

**Table 4:** Foraging mode and echolocation call frequency of adult bat species reported to be captured in spider webs (arranged in order of increasing peak frequency).

Bat species	Foraging mode	Echolocation callpeak freq [kHz]	Source	report #
*Cyttarops alecto*	Aerial insectivore	36	[143]	10
*Centronycteris centralis*	Aerial insectivore	41	[143]	12
*Chalinolobus gouldii*	Aerial insectivore	41	[144]	41
*Nycticeinops schlieffeni*	Aerial insectivore	42	[145]	46
*Pipistrellus abramus*	Aerial insectivore	45	[147]	26–27, 29–30
*Saccopteryx bilineata*	Aerial insectivore	45 and 47	[143]	1, 21
*Pipistrellus pipistrellus*	Aerial insectivore	46	[146]	52
*Myotis nigricans*	Aerial insectivore	54	[148]	2, 22–24
*Pipistrellus hesperus*	Aerial insectivore	62	[149]	49
*Tylonycteris pachypus*	Aerial insectivore	65	[73]	37
*Neoromicia nana*	Aerial insectivore	69	[145]	48
*Nyctophilus gouldi*	Gleaning insectivore	72	[144], [150]	43
*Rhynchonycteris naso*	Aerial insectivore	98	[143]	7, 14–16
*Rhinolophus cornutus*	Gleaning insectivore	110	[80], [151]	35
*Glossophaga sp.*	Nectarivore	117	[152]	20
*Hipposideros pomona*	Gleaning insectivore	133	[137], [153]	31
*Hipposideros ater*	Aerial insectivore	160–164	[154]	39, 44

The species name *Vespertilio irretitus* has changed to *Pipistrellus abramus* (see [32]).

### Size Ratio of Web-building Spiders Versus Bats

Bats trapped in spider webs are usually small-sized (3–8 g adult weight; Table 3), whereas the spiders capable of overpowering bats are giant orb-weavers (∼0.5–7 g weight; Table 2). The largest bat species reported to have been captured in spider webs were an adult male *Nyctophilus gouldi* (a species with a known adult weight of 11 g) and a *Chalinolobus gouldii* of unknown age (a species with a known adult weight of 15 g; Table 3). The tensile strength and elasticity of silk produced by large nephilid and araneid orb-weavers is high (e.g. [95]), enabling such “high energy absorbing webs” to retain flying vertebrates whose weight exceeds by far the spiders’ own weight. Large orb-weaving spiders are generally capable of trapping and killing prey that is much larger than themselves [96]. For instance, a *Nephila* sp. was reported to have captured a 30–35 g bird, a Lewin’s Honeyeater, *Meliphaga lewinii* [24]. Furthermore it has been reported that an 18 g Grasshopper Sparrow, *Ammodramus savannarum,* was trapped in a spider web in New York [24], while a 90–110 g Black-faced Cuckoo-Shrike, *Coracina novaehollandiae,* was ensnared in a spider web in Australia [24]. The exact circumstances under which these relatively heavy birds were trapped in spider webs are not reported. Flying vertebrates of fairly large size might be retained if such an animal crashes into an entire aggregation of large, strong *Nephila* webs.

## Discussion

### Are the Witnessed Incidences Real Predation Attempts?

It is arguable whether all incidences reported in this paper are real predation attempts or whether some are just deaths by web ensnarement without the active involvement of the spider (non-predation deaths). Begon et al. [97] define the term predation as follows “…Predation, put simply, is consumption of one organism (the prey) by another organism (the predator), in which the prey is alive when the predator first attacks it…” With other words, the definition of predation implies that a prey item must have been killed and eaten by the predator.

With regard to bat-eating theraphosids photographed in the Neotropics and in India, the actual killing of the bats was not witnessed. However, it has been proven by means of observations in captivity that large theraphosids are capable of killing bats. This is shown in a YouTube video where a *Grammostola rosea* (subfamily Theraphosinae) is killing a small bat offered to it in a cage environment (www.youtube.com/watch?v=kmGEoaHBhew; this report was not included in Table 1 because it occurred in captivity). The G*rammostola rosea* shown in this video was identified for us by R. West (pers. comm.). Theraphosid venom has been proven to be active on small mammals [20]. One can therefore assume that theraphosid predation on bats is taking place in the field as well and that the incidences witnessed in the Neotropics and in India may have been cases of predation and not scavenging.

With regard to the sparassid *Heteropoda venatoria,* reported to have killed a small bat in India [36], the situation is somewhat different. Here the spider did catch and kill the bat, but the sequence of behavioral units naturally displayed during a predation event - starting with the attack of the prey and ending with the cessation of feeding - was disrupted as the observer interfered by capturing spider and bat and placing them into a glass jar. As it appears, the dead bat was subsequently not preyed upon (non-predation death). The spider in question might have preyed on the bat had it not been disturbed by the observer. *Heteropoda* spp. often occur in high abundance in close proximity to bat roost sites in places like caves, buildings, and trees [63], [98]. Especially in caves in Southeast Asia, large-sized *Heteropoda* spp. cohabit with different species of bats [63], [65]–[66], [69]. Large-sized *Heteropoda* spp. are powerful enough to subdue small bats, especially if one takes into consideration that they possess impressive chelicerae, potent venom against small vertebrates, and the ability to move at high speed [17], [19], [64]. Furthermore, these spiders show morphological characteristics suggesting that they may be highly adapted to climb vertical surfaces and walk on cave ceilings [69], [99]. The spiders may climb cave walls and ceilings to catch perching bats or they may search for bat pups fallen from the roost to the floor. It is readily imaginable that the *Heteropoda* spp. supplement their staple diet, made up of arthropods (i.e., in particular cave crickets, cockroaches, arachnids, and centipedes; e.g. [64], [69]), by the occasional catch of a small bat. One way to find out whether *Heteropoda* spp. feed on bats would be to video record the behavior of these spiders at locations where they forage in close proximity to bat colonies. In 2006 (April to December), such a study was undertaken at several pipistrelle roost sites in the Christmas Islands, Australia; there, five video cameras were deployed for a total of 663 trap nights [98]. Sparassid spiders, *Heteropoda venatoria*, were the most frequently recorded potential predators on the trunks of the roost trees, with 43 individual records. The video recordings, however, did not reveal any of these spiders preying on bats, disturbing them or entering their roost sites. Currently we cannot confirm whether *Heteropoda* spp. are habitually engaged in bat predation or whether the incidence witnessed by Bhattacharya [36] was just a chance occurrence. Further studies on possible interactions between sparassid spiders and small-sized bats are therefore of considerable interest (also see [100]).

The Neotropical orb-weaving spider *Eriophora fuliginea* has been observed to kill and eat small bats that got entangled in its webs [8], [37], [101]. When a bat got caught in a web, the spider immobilized the bat by attack-wrapping and subsequently biting it [101]. Following this, the spiders fed on the dead bats for many hours (D.E. Wilson, pers. comm.). The incidences of bats being caught, killed, and eaten in webs of *Eriophora* spp. are without any doubt predation events.

Likewise, the orb-weaving spider *Nephila pilipes*, occurring in Asia and Australia, has many times been observed to attack, kill and eat small bats. Contrary to *Eriophora*, *Nephila pilipes* immobilizes its prey by always first biting and subsequently wrapping it [38], [102]. The immediate attack bite of *Nephila* towards bats has been witnessed by two authors [11], [103], whereas the consumption of bats was repeatedly observed/photographed ([39]–[40], [104]; C. Fabro, pers. comm.; C.S.K. Liu, pers. comm.). There are several reports where *Nephila pilipes* was seen killing and eating small birds as well (e.g. [28]). Thus, the behavior of *Nephila pilipes* towards small bats (catching, killing, and eating them), clearly complies with the definition of predation.

The Neotropical orb-weaving spider *Nephila clavipes* has been witnessed catching bats quite frequently (9 reports), but in none of these cases was it seen biting, wrapping or eating a bat. Likewise, birds trapped in the webs of this spider species were apparently not consumed [22]–. Only once has a Neotropical *Nephila* been observed biting a bird, but without subsequent consumption of the prey [22]. It has been suggested that *Nephila clavipes* might be unable to deal with large, aggressive prey such as bats and birds [24], [27]. If this latter assumption is true, then the captures of bats in the webs of *Nephila clavipes* would be cases of non-predation deaths (the bats dying of exhaustion, starvation, dehydration, and/or hyperthermia). The two European incidences where bats were killed in spider webs without the spiders feeding on them must be considered to have been cases of non-predation deaths as well.

In conclusion, some of the bats entangled in spider webs are actively killed and consumed by the spiders (i.e., predation), whereas in other instances the entangled bats are not consumed by the spiders (i.e., non-predation deaths). In several of the incidences, where dead bats were found suspended in spider webs, it could not be determined whether predation had taken place because of the bats’ desiccated condition (e.g. [44]).

### How Frequent are Bat Catches by Spiders?

We conducted a survey of a large number of chiropterologists and arachnologists who had conducted extensive field investigations in the tropics/subtropics to find out how many of them have ever witnessed a spider catching a bat. A bat trapped in a spider web is a fairly conspicuous sight that an experienced biologist will hardly overlook, especially since it takes a spider several hours to handle a bat prey. Only very few bat scientists (e.g. [8], [30], [105]) have ever witnessed an incidence of a bat caught by a spider, while many others who also spent decades in the field (e.g., B. Fenton, pers. comm.) have never seen this. Likewise, very few orb-weaving spider experts (e.g. Robinson [101]) have witnessed spiders catching bats. The 52 incidences reported in our review, which refers to a time period of more than 100 years (starting with Cantor’s report in 1842 [106]), is very low if this figure is compared to estimates of bat mortality attributable to the bats’ chief natural enemies. For instance, Speakman [2] estimated that ∼200,000 bats per year are killed as a result of predation by owls and kestrels in Great Britain alone. The fact that bat catching by spiders has been witnessed so infrequently suggests that this type of bat fatality is extremely rare. This is surprising because in tropical/subtropical areas the millions of huge webs of *Nephila* spp. and *Eriophora* spp. stretched across the bats’ flight paths pose an enormous risk to bats (especially in locations where such orb-webs are aggregated) and, actually, one would expect large numbers of bats being killed each night. It appears that bats are evolutionarily well-adapted to detect and avoid spider webs [107]–[108]. Bats are likely capable of detecting spider webs by means of echolocation. Though single silk strands of spiders (∼0.002–0.005 mm in diameter; [109]) are probably below the detection threshold of echolocating bats (∼0.16 mm for the aerial insectivore *Eptesicus fuscus*; [110]; H.-U. Schnitzler, pers. comm.), the webs as a whole, often containing additional conspicuous, densely-woven silk decorations or silk barriers [54], [107], may present themselves as tangible objects that presumably bounce back echolocation calls emitted by the bats with enough intensity to be detectable by the bats [107]. Not only the webs, but also the spiders (in particular the huge adult females of *Nephila* spp. and *Eriophora* spp. staying at the hub of the web all night long) presumably generate strong echoes that the echolocating bats should be able to detect, advertising to them the presence of obstacles that need to be avoided. In a study in Australia, Schultz & Wainer [87] captured more than 1200 bats (representing eleven species) and checked them for the presence of spider web fragments around their wing membrane and/or on the body fur. These authors found that the vast majority of bats did not have any traces of spider web fragments attached to their body, strongly confirming the above-mentioned hypothesis that bats are able to largely avoid encounters with spider webs. This is also confirmed by a study conducted in British Columbia where no spider web material was found attached to the body of two species of *Myotis* bats trapped in mist nets [83]. However, even if some bats collide with spider webs, a considerable proportion of these bats may be able to elude ensnarement by the following reasons: First, only large webs from a limited number of giant orb-weaving spider species (especially *Nephila* spp. and *Eriophora* spp.) are strong enough to withstand the tremendous kinetic energy of a flying bat without breaking. Such webs that intercept heavy and fast flying prey have been termed “high energy absorbing webs” (*sensu* Craig [90]). If bats strike a smaller web (i.e., “low energy absorbing webs”, designed to intercept light and slow flying prey), the bats fly right through it, leaving behind a damaged or destroyed web (also see [87], [89]). Second, only small bats can be retained in orb webs. If a larger-sized bat strikes a web of any size, the kinetic energy is too high to be absorbed and the web will break. Thus, larger-sized bats will usually fly right through a web, leaving behind a big hole. The same happens when larger-sized birds fly through spider webs [54]. Third, even if small bats get entangled in large webs, a certain percentage of violently struggling bats is able to escape (D.E. Wilson, pers. comm.). The same is true for small birds that may get temporarily entangled in spider webs but are often able to free themselves after a short time of struggling [23]. It should be noted that various species of bats behave differently when trapped in a web, resulting in differing chances to escape. This is known from experience with trapping bats in mist nets, which may be looked upon as “huge artificial spider webs”. For instances, *Rhynchonycteris naso* does not defend itself if trapped in a net, whereas other sympatric bats of comparable size (e.g., *Rhogeessa io*) struggle violently (M. Knörnschild, unpubl. data). Accordingly, chances for a *Rhynchonycteris naso* to escape from a large spider web are slim compared to other bat species. Adaptation for escape from spider webs also exists in various taxa of flying insects (e.g. [111]–[112]).

Nevertheless, some bats get caught and killed in spider webs. Such cases might be considered to be “accidents”. Why do such accidents happen? There may be several possible reasons for this:

First, it is noteworthy that the majority of the identifiable bats (65% of reports) that accidentally crashed into spider webs echolocate at frequencies of only ∼36–72 kHz (Table 4). It could be that the echolocation calls of these aerial insectivorous species (e.g., *Pipistrellus* spp. and *Myotis nigricans*) are less well-adapted to detecting spider webs compared to the high frequency echolocating species (G. Jones, pers. comm.). Bats such as the pipistrelles and *Myotis nigricans* are relatively fast flying and may not always be able to avoid the webs if detected only at close range (G. Jones, pers. comm.). Thus, though we hypothesize that most bats are able to largely avoid encounters with spider webs (see above), accidents do happen and bats echolocating with lower frequencies might be engaged in these accidents with a higher likelihood. Second, Griffin [113] theorized that bats flying in familiar territory rely heavily on spatial memory and not on echolocation. This might be the case when bats fly in proximity to the roost or when they use “flyways” from the roost to the hunting ground and *vice versa* [114]. In such situations of heavy reliance on spatial memory, the bats might not notice a spider web until they have already hit it. Indeed several incidences reported in this paper (27% of reports) occurred while the bats were flying in proximity to their roost (i.e., buildings, caves, and forest trees [30], [44]; M. Knörnschild, unpubl. data) and thus in spots where the bats presumably did not rely heavily on echolocation. Third, some bats found entangled in spider webs were juveniles or inexperienced subadults (reports # 7, 15–16, 20–23, and 50). As unskilled flyers, young bats may be more susceptible to accidents than adults [115]–[117]. A special case was the incidence observed in Florida where bat pups of the Southeastern Brown Bat got entangled in spider webs after falling from the roost [43]. Bat pups falling from the roost frequently become prey of various predators [7]. Fourthly, some bats may get captured when they crash into an aggregation of large orb-webs. Large orb-weavers of the genus *Nephila* tend do aggregate under circumstances of high prey availability [49], [118]. Blackledge et al. [119] states: “…While reducing the overall number of prey that might be intercepted, these spiders gain access to larger insects that would normally break through a web as the insects either ricochet off or slow down as they pass through exterior webs…” This is exemplified by Sue Churchill’s observation at Tolmer Falls, Australia, where an adult *Hipposideros ater* had apparently got partially tangled in one web and then fallen into the other webs before being fully caught (report # 44). Fifthly, bats that habitually glean insects from spider webs while hovering in front of them [120] may sometimes get entangled after accidentially striking the web. This evidently happens despite the fact that these bats, echolocating at high frequencies (>100 kHz start frequency [81], [88]), are thought to be evolutionarily adapted to avoid collision with spider webs. The *Rhinolophus cornutus orii* captured in a *Nephila* web in Japan (Fig. 2I) may exemplify an incidence of this type (report # 35). Bats from this genus sometimes strike spider webs, evidenced by the fact that 4 out of 58 specimens of *Rhinolophus megaphyllus* captured in harp traps in Australia had spider web fragments attached to their wing membrane and body fur [87]. The spider webs in this latter study were obviously not strong enough to withstand the bats’ kinetic energy without breaking (“low energy absorbing webs”). Sixly, during periods of food scarcity, weakened small bats might be unable to free themselves if trapped in spider webs, even if the webs are not as strong as those spun by tropical large orb-weavers. The incidences witnessed in Germany and England, where small pipistrelle bats were killed in webs without any involvement of the spiders, might present examples for this type of non-predation deaths.

At the present time nothing is known about the frequency with which theraphosids and sparassids catch bats. The cryptic life habits of these predominantly nocturnal hunters make them difficult animals to study. Likewise, nothing is known about the frequency of predation on bats by pisaurids.

### Are Nephilid Webs at Cave Entrances a Threat to Cave-roosting Bats?

In the tropics, huge nephilid orb-webs (genera *Nephila* and *Nephilengys*) sometimes block the entrances to bat caves ([61]; C. Dietz, pers. comm.). Such cave entrance inhabiting nephilid populations have been discovered in East and South East Asia as well as in the Neotropics. So far it is unknown to what extend cave-roosting bats flying back and forth between caves and foraging areas are able to detect and avoid these webs. Since it is hypothesized that the bats might navigate by spatial memory while passing through cave entrances [121], it is conceivable that some of them may crash into nephilid webs within the cave’s entrance zone, given the fact that in some areas they leave caves at dusk in gigantic swarms. Monitoring nephild webs at cave entrances by means of video recording devices could bring an answer to this question.

### How Important is Chiropterophagy from a Point of View of Spider Nutritional Ecology?

All five groups of bat-catching spider taxa (Nephilidae, Araneidae, Theraphosidae, Sparassidae, and Pisauridae) are known to be predominantly predaceous on insects [14], [53], [64], [69], [101]. With regard to large-sized theraphosids, sparassids, and pisaurids, their feeding behavior in the field has not been thoroughly investigated and one cannot currently judge whether predation on bats is of significance to them from a feeding ecological point of view.

Our current knowledge of orb-weaver feeding ecology suggests that these spiders depend on flying insects as main prey, whereas bats and also birds occasionally entangled in spider webs might be considered to be by-catch [45]. It can take a large orb-weaver many hours to consume a bat or bird prey ([26], [28], [30]; D.E. Wilson, pers. comm.) indicating that spiders might extract a substantial amount of energy while feeding on such a large prey. Based on field observations in Papua New Guinea, Robinson & Robinson [53] estimated the average capture rate of *Nephila pilipes* at ∼0.19 g wet weight prey killed per spider per day (which corresponds to ∼0.015 g dry weight food ingested per spider per day). According to Higgins [46], the average prey capture rate of the significantly smaller-sized *Nephila clavipes* in Texas was ∼1.5–2.5 times lower than the estimate by Robinson & Robinson [53]. Thus, the catch of a ∼2 g bat yields a *Nephila pilipes* a potential prey biomass that is about 10 times the average daily prey catch.

In recent years, the idea has been proposed that the occasional catch of large, energetically rewarding prey may be essential in order to fulfil the reproductive needs of large orb-weaving spiders (“rare, large prey” hypothesis; see [122]–[123]). While large orb-weavers such as *Nephila* spp. capture predominantly small insects of little energetic value, they derive the bulk of their energy from a few rare, large prey items (see [46], [49], [53], [122]). In this context “rare, large prey” encompasses large insects (e.g., cicadas, moths, coleopterans, orthopterans, and odonates) as well as small flying vertebrates (bats and birds). In our opinion, the examples of bat-eating orb-weavers reported in this paper are consistent with the "rare, large prey" hypothesis, though one may object to this given the rarity of such events.

## Supporting Information

File S1 **Detailed Reports Description.**
(DOCX)
