# Supplementary material for: Bat Predation by Spiders
Source: PLoS One. 2013 Mar 13;8(3):e58120. doi: 10.1371/journal.pone.0058120 (PMC3596325; doi:10.1371/journal.pone.0058120)
Supplement: File S1 — Detailed Reports Description. (DOCX) [file pone.0058120.s001.docx]

**SUPPORTING INFORMATION FILE S1**

**Bat Predation by Spiders - Martin Nyffeler & Mirjam Knörnschild**

**DETAILED REPORTS DESCRIPTION**

**HUNTING SPIDERS**

There are several reports on bats being eaten by hunting spiders. Liat (1964) and Kaston (1965) fed bats to tarantulas (Theraphosidae) in captivity. However, there are also reports of theraphosids and other hunting spiders eating bats in the field.

**South America**

**# 1) -** In November 1994, an adult female *Avicularia urticans* (subfamily Aviculariinae) was photographed eating a Greater Sac-winged Bat *Saccopteryx bilineata* (Emballonuridae) on the side of a palm tree on the fringe of a tropical rainforest near the Rio Yarapa, Peru (Conniff 1996; Rick West, pers. website; Fig. 2A). Being near the rivers and creeks, this was an ideal nocturnal foraging habitat for insectivorous bats. Spiders of the genus *Avicularia* are arboreal. The spider in question was ~5-6 cm in body length (cephalothorax plus abdomen).

**# 2) -** A similar incidence of a medium-sized theraphosid feeding on a small bat was photographed by George Schmid in August 2006 in eastern Ecuador (G. Schmid, flickr image hosting website: <http://www.flickr.com/photos/85015297@N00/224189793/>); here an *Avicularia* sp. was feeding on a small bat (Black Myotis, *Myotis nigricans*; Vespertilionidae) in close proximity to a bat roost. *Saccopteryx bilineata* and *Myotis nigricans,* the two bat species eaten by *Avicularia* spp., are both common Neotropical bats which often roost on or in rainforest trees (Wilson 1971; Young 1972).

**# 3) -** In the early 1980s, a large tarantula, *Lasiodora parahybana* (subfamily Theraphosinae), was photographed eating a bat on the forest floor (Rick West, pers. website). The bat species was not identified. This incidence was discovered during a trip from Joao Pessoa to the higher elevations of the Caatinga-thorny scrub forest in northern Paraiba State, Brazil (R. West, pers. comm.). *Lasiodora parahybana* is an extraordinarily large species that can reach a legspan of 20-25 cm (World Association of Zoos and Aquariums 2011).

**Asia**

**# 4) -** A Reddish Parachute tarantula spider (*Poecilotheria rufilata;* subfamily Poecilotheriinae) was reported killing and eating a Kelaart’s Pipistrelle bat (*Pipistrellus ceylonicus;* Vespertilionidae) in the Chinnar Wildlife Sancturaly, Kerala, India, in November 2011 (Das et al. 2012). The spider caught the bat approximately ten meters away from its roosting site in a concrete building. It had a total body length of 65 mm and was estimated to weigh between 28-85 grams.

**# 5) -** A hunting spider, *Heteropoda venatoria* (Sparassidae) was photographed catching and killing a pipistrelle bat in a shed in a neighbouring village of Kolkata (formerly Calcutta) located in tropical India (Bhattacharya 1941). The bat was screaming loudly and flapping its wings, while the spider was gripping it by the neck with its powerful chelicerae. This was going on for about 15-20 minutes, after which time Bhattacharya confined the two combatants in a glass jar, where they were left alone over the night. In the morning the bat was dead, but the spider had apparently not fed upon it.

**North America**

**# 6) -** In late June 1991, Phil Clem (University of Charleston, West Virginia) and Virgil Brack (formerly Indiana State University) witnessed an incident where a large *Dolomedes triton* was attacking an immature bat below a bridge in Parke County, Indiana, USA. The spider was on top of the bat (a prevolant Northern Myotis, *Myotis septentrionalis;* Vespertilionidae*)* apparently biting it on the nape while the bat, emitting distress cries, was trying to free itself. This spider-bat encounter was observed for about three minutes after which the spider escaped into the water, probably frightened by the presence of the photographing observers (P. Clem & V. Brack, pers. comm.). Subsequently the bat (weighing 2.3 g) was examined without revealing any noticeable bite marks or adverse bite effects. *Dolomedes triton* is a species weighing on average ~1 g that is capable of overpowering prey of up to 4.5 times its own weight and whose venom is very effective in killing small vertebrate prey (Bleckmann & Lotz 1987; Suter & Gruenwald 2000). Whether the spider would have killed and eaten the bat (had it not been frightened away) remains unknown.

**WEB-BUILDING SPIDERS**

**South and Central America**

**# 7) -** In November 2007, Marjorie Weber (Cornell University, USA), Adriana Bravo (American Museum of Natural History, USA), and Sam Barnard (Colorado College, USA), stationed at the Los Amigos Biological Station in Madre de Dios, Peru, made a trip to a nearby oxbow lake. The site is a palm swamp forest in the middle of lowland tropical rainforest. The scientists found three bats trapped in a large *Nephila clavipes* web built over the water of the lake where the bats flew (M. Weber, pers. comm.). Possibly the bats (one newly volant young and two adult individuals) were resting together on a nearby daytime roost close to the water and flew into the web after having been frightened by the visitors (compare Plumpton & Jones 1992). The bats were identified by Alfred Gardner (USGS-Patuxent Wildlife Research Center, National Museum of Natural History), Mirjam Knörnschild (Institute of Experimental Ecology, University of Ulm), and Adriana Bravo (American Museum of Natural History) as the Proboscis Bat, *Rhynchonycteris naso* (Emballonuridae). The volant juvenile was photographed by Sam Barnard (Fig. 2G). The next day, when the scientists returned to this site, the bats were still hanging in the web (M. Weber, pers. comm.). Unfortunately the behavior of the spider towards the bats was not recorded.

**# 8) and # 9) -** Two incidences of bats of unknown species captured in spider webs were witnessed by Dario Hernando Gutierrez, a biologist affiliated with the Museo Aranas de Colombia, Bogota. The first incidence occurred in 1998 in an area of tropical secondary forest outside of Bogota. There, a dead bat was found ensnared in the web of a female *Nephila clavipes* suspended between a mango tree and shrub vegetation about 2 m above the ground (D.H. Gutierrez, pers. comm.). The second incidence occurred in 2003, when a bat carcass was found in the web of a female *Nephila clavipes* built between shrubs approx. 1.5 m above the ground in shrubland near Bogota. Several tiny kleptoparasites of the theridiid subfamily Argyrodinae were present in this second web (D.H. Gutierrez, pers. comm.).

**# 10) -** In October 1972, an adult male Short-eared Bat, *Cyttarops alecto* (Emballonuridae) was found entangled in a spider web by the taxonomists H. Chiriví, R. Cooper und A. Díaz (Ochoa et al. 1994). The observation took place in the Columbian Amazon near Calderón. The spider was not identified. It was not stated whether the bat was already dead when being collected. The skin of the bat was undamaged, suggesting that the spider had not fed on it.

**# 11) -** In May 2009, a dead bat was found entangled in the web of a female *Nephila clavipes* in Guatemala (S. Bloomquist, pers. comm.). This incidence was photographed by Sam & Samantha Bloomquist (Fig. 2H) in a tropical rainforest in the middle of the Rio Dulce River Canyon near Livingston. It was not possible to identify the bat based on the photograph.

**# 12) -** In the mid-1990s, a dead bat was found entangled in an orb-web in Belize (C. Farneti-Foster, pers. comm.; Fig. 2C). The web was suspended between citrus trees planted along tropical rainforest. By means of the photograph the bat was identified by Alfred Gardner (USGS-Patuxent Wildlife Research Center, National Museum of Natural History) to be most likely a Thomas’s Shaggy Bat, *Centronycteris centralis* (Emballonuridae). The orb-weaver was probably an *Eriophora* sp. (Araneidae).

**# 13) -** Another incidence - this time occurring in Southern Belize - has been documented in a film produced by the National Geographic Society; the film shows an araneid orb-weaving spider immobilizing a small bat by attack-wrapping after the bat had crashed into the spider’s web (<http://video.nationalgeographic.com/video/player/animals/bugs-animals/spiders-and-scorpions/spider_orb_kills_bat.html>).

**# 14) -** In July 2005, an adult Proboscis Bat, *Rhynchonycteris naso* (Emballonuridae), was found entangled in a web of *Argiope savignyi* (Araneidae) at the La Selva Biological Station in the Caribbean lowlands of northern Costa Rica (M. Knörnschild, unpubl. data; Timm & Losilla 2007). This area is dominated by tropical rainforest. The spider web (~30-40 cm in diameter) was built on the outside of a station building, situated in the immediate vicinity (about 10 m air-line distance) to the Rio Sarapiqui river and only ca. 1.5 m away from a *Rhynchonycteris naso* colony. *Argiope savignyi* is a largely diurnal spider with a body length of ~10-14 mm that occurs from Mexico to Bolivia (Platnick 2012). During the night, Proboscis Bats use the river as foraging habitat. The spider was apparently feeding for several hours on the dead bat which was totally wrapped in silk (Timm & Losilla 2007). This incidence was photographed by Mirjam Knörnschild (Fig. 2B) and by Timm & Losilla (2007).

**# 15) -** A similar incidence of a *Rhynchonycteris* *naso* being captured in an *Argiope savignyi* web was witnessed in 2006 (M. Knörnschild, unpubl. data). In this second incidence the dead bat was a volant juvenile and the web (~30-40 cm in diameter) was again built on the outside of one of the buildings of the La Selva Biological Station in close proximity to the Rio Sarapiqui river and to a *Rhynchnycteris naso* colony.

**# 16) -** In 2008, a subadult *Rhynchonycteris naso* was caught in the web of a *Nephila clavipes* after the bats were disturbed in their day-roost (M. Nagy, pers. comm.). The web was built in close vicinity to a *Rhynchnycteris naso* colony located in an uninhabited building of the La Selva Biological Station in Costa Rica. The bat was immediately freed by the observer and released.

**# 17) -** A dead bat was found entangled in the web of a *Nephila* *clavipes* in La Sirena, Corcovado National Park, Costa Rica (H. & G. Unger, pers. comm.). This incidence was photographed by Harald and Gisela Unger (Fig. 2D). Based on the photograph, the bat was identified by Alfred Gardner (USGS-Patuxent Wildlife Research Center, National Museum of Natural History), Don Wilson (National Museum of Natural History, Smithsonian Institution), and Richard Laval (Programa para la Conservación de Murciélagos de Costa Rica, Monteverde) to be a *Myotis* sp. (Vespertilionidae).

**# 18) -** In mid 2010, Cassidy Metcalf posted a photograph (Fig. 2E) taken in Tortuguero National Park, Costa Rica (<http://www.flickr.com/photos/cassinafrica/4832280112/in/photostream>), which depicts a small bat entangled in the web of an ecribellate orb-weaving spider, possibly an *Eriophora* sp.. The spider had created a web over a walkway that spanned approx. 2.5 meters in distance, from one palm-thatched roof to another (C. Metcalf, pers. comm.). The spider had its mouth pressed against the dead bat, suggesting that is was feeding on it.

**# 19) -** At dawn on 28 August 2012, a medium-sized bat got entangled in a *Nephila clavipes* web at the Biological Station La Sirena in the Corcovado National Park, Costa Rica. The biologists Marcos Mallo und Carmen Díez observed that the bat was able to escape from the web after less than a minute. While it was struggling to free itself, two other bats were circling the spider web (M. Mallo & C. Díez, pers. comm.).

**# 20) -** Another incident has been documented in a film produced in Costa Rica by gettyimages (<http://www.gettyimages.ch/detail/video/close-up-brown-bat-struggling-in-spider-web-with-stock-videomaterial/131557930>; <http://www.gettyimages.ch/detail/video/close-up-brown-bat-struggles-in-spider-web-stock-videomaterial/131558090>). A subadult long-tongued bat (*Glossophaga sp.;* Phyllostomidae) was entangled in a spider web in Costa Rica. Despite the fact that the spider itself was not seen in the film, it could be identified as *Nephila clavipes* based on the web’s specific structure and yellow color (S. Zschokke, pers. comm.). The bat repeatedly tried to free itself but to no avail. It is unclear whether the bat finally escaped, died or was predated upon by the spider.

**# 21) -** In August 2012, it was witnessed how a subadult Greater Sac-winged Bat, *Saccopteryx bilineata* (Emballonuridae) got entangled in the web of a *Nephila* *clavipes*. This incidence was witnessed near the Smithsonian Tropical Research Station on Barro Colorado Island, Panama Canal Zone (M. Eckenweber, pers. comm.). The bat was immediately rescued by the observer, cleaned from web material sticking to its body, and released.

**# 22) and # 23)** **-** Robinson et al. (1971) and Wilson (1971) observed small bats being killed in webs of the large orb-weaving spider *Eriophora fuliginea* (Araneidae). These scientists have also flown live subadult Black Myotis, *Myotis nigricans* (Vespertilionidae), into the webs of *Eriophora fuliginea* (see below). The observations and experiments were made at web sites in the laboratory clearing of the Smithsonian Tropical Research Station in Barro Colorado Island, Panama Canal Zone, an area surrounded by lowland tropical rainforest. A colony of *Myotis nigricans* inhabited the attic of the laboratory building. When a bat got caught in a spider web, the spider immobilized the bat by attack-wrapping and subsequently biting it (see Robinson et al. 1971).

**# 24) -** A photograph of a dead *Myotis nigricans* (Vespertilionidae) entangled in the web of an *Eriophora fuliginea* in Panama is included in Levi (1970).

**# 25) -** The attack-wrapping of small bats by such araneid spiders can be seen in a Nature (PBS) film entitled “Selva Verde: The Green Jungle” which had been made in Central America with M.H. Robinson as scientific advisor (<http://www.youtube.com/watch?v=4-BhN5F42SE>). Wilson (1971) states: “…Numerous experiments showed that even if the spider merely cuts the bat out of the web, the bat probably dies due to the entangling pieces of web which prevent its flight…” In cases where spiders succeeded in killing a bat they afterwards fed on it for several hours (D.E. Wilson, pers. comm.).

**Asia**

**# 26) and # 27) -** Incidences of bat-catching spiders have been reported from the Island of Zhoushan, south of Shanghai, China (Cantor 1842). Zhoushan is an island with subtropical monsoon marine climate, characterized by the absence of forests. According to Cantor (1842), Japanese Pipistrelle, *Pipistrellus abramus* (Vespertilionidae) were frequently entangled in the webs of two large orb-weaving spiders, *Araneus bilineatus* and *Araneus heraldicus* (Araneidae). Apparently the trapped bats were not preyed upon by the spiders (Cantor 1842).

**# 28) -** Kershaw (1905) reports an observation from Southern China (somewhere in the subtropical Macau-Hong Kong area) where a *Nephila pilipes* was devouring a small bat caught overnight in its web.

**# 29) -** Hill et al. (1982) reported an incidence where a *Nephila pilipes* was eating the remains of a Japanese Pipistrelle, *Pipistrellus abramus* (Vespertilionidae) caught in its web. This observation was made in the Tai Po Kau forest, a regenerating subtropical forest, in Hong Kong.

**# 30) and # 31) -** Gary Ades, Kadoorie Farm & Botanic Garden Hong Kong, informed us about anecdotal reports of bats being caught in webs of *Nephila pilipes* in Hong Kong. Usually these reports refer to the Japanese Pipistrelle, *Pipistrellus abramus,* but once a Pomona Leaf-nosed Bat, *Hipposideros pomona* (Hipposideridae) was reported to have been ensnared in a *Nephila pilipes* web (G. Ades, pers. comm.).

**# 32) -** In September 2011, Carol S.K. Liu from AFCD Hong Kong (Agriculture, Fisheries and Conservation Department) photographed a dead vespertilionid bat entangled in the web of a female *Nephila pilipes* in the Aberdeen Country Park, Hong Kong (Fig. 2L). The bat was identified by Marc Holderied (University of Bristol) and Tigga Kingston (Texas Tech University) to be a species belonging to the family Vespertilionidae. In other photographs not included in this paper one can also see two tiny red-coloured kleptoparasites of the theridiid subfamily Argyrodinae apparently feeding on the bat.

**# 33) -** In August 2009, an anonymous blogger posted a photograph taken on Hong Kong Island of a dead bat caught in the web of a female *Nephila pilipes* on the internet (<http://www.brezhnev.net/photos/hk/batspider.jpg>).

**# 34) -** In September 2008, a bat was caught in the web of a *Parawixia dehaani* (Araneidae) in a building located in the Tai Po Kau forest, Hong Kong (G. Ades, pers. comm.). *Parawixia dehaani* is a nocturnal orb-weaver of approx. 2 cm body length which captures predominantly moths in its vertical webs (Koh 2000). The bat was disfigured by the spider.

**# 35) -** On 19 September 2007, the *Asahi* Newspaper, Japan, published a short report (based on observations by Yasunori Maezono, Kyoto University) of a bat being caught in the web of a female *Nephila pilipes* on Amami-Oshima Island, Japan. This island is covered with subtropical forests where *Nephila pilipes* builds large webs (1m in diameter) along forest trails. The dead bat was identified as Little Japanese Horseshoe Bat, *Rhinolophus cornutus orii* (Rhinolophidae). The spider bit the neck of the bat and appeared to consume body fluid (Fig. 2I).

**# 36) -** In Halong Bay, Vietnam, it was witnessed how a large *Nephila pilipes* was apparently feeding on a dead insectivorous bat. This observation was made in a sparse pine forest with fern undergrowth located in the tropical climate zone. A photo of this incidence was posted in July 2006 on the internet by blogger Padraig Larkin (<http://www.thebogman.org/travelog/uploaded_imagines/P7070128-776583.JPG>).

**# 37) -** During field studies conducted at the University of Malaysia’s field station in Ulu Gombak, Selangor, West Malaysia, a small Lesser Bamboo Bat, *Tylonycteris pachypus* (Vespertilionidae) was found entangled, but otherwise uninjured in the web of an unidentified web-building spider (Medway 1972). Obviously the bat crashed into the spider web a short time before the observers arrived; otherwise it would have died from hypothermia and dehydration. The station is situated in a reserve of tropical secondary lowland rainforest where the bats roost in hollow bamboo internodes.

**# 38) -** Sherriffs (1934) reports an incidence from Kandaloya, Dolosbage, Sri Lanka, located in the tropics, where a *Nephila pilipes* was apparently devouring a small bat caught in its web. Kandaloya is a large agricultural landholding with associated buildings.

**Australia and Papua New Guinea**

**# 39) -** On 24 January 2009, Carmen Fabro (pers. comm.) photographed a small bat hanging in the web of an adult female *Nephila pilipes* (Fig. 2J-K). The pictures were taken at the top of the Cockatoo Hill, a resort surrounded by Daintree tropical rainforest near Cape Tribulation, Queensland. The spider web, stretching across a foot path, was attached to palms on one side and lipstick palms on the other side, about 10-15 m away from the nearest building. It was hanging there for a couple of weeks. This agrees with the observations by Robinson & Robinson (1976) who stated that adult female *Nephila pilipes* may stay in one place for long periods of time. Based on photographs, the bat was identified by a group of Australian bat scientists (including Kyle Armstrong, Mike Craig, Chris Pavey, Terry Reardon, Martin Rhodes, and Christopher Tidemann) as belonging to the superfamily Rhinolophoidea. The majority of experts identified the bat as Dusky Leaf-nosed Bat, *Hipposideros ater* (Hipposideridae), whereas a few experts suggested that it could be either *Hipposideros ater* or a juvenile Smaller Horseshoe Bat, *Rhinolophus megaphyllus* (Rhinolophidae). As is seen in the photograph, the spider pressed its mouth against the dead, wrapped bat, indicating that it was feeding on it. A *Nephila pilipes* male also present may have been feeding on the bat as well (Fig. 2K). The following morning there was a big hole in the web; the bat had disappeared. A bird or larger bat might have taken the bat carcass during the night (C. Fabro, pers. comm.).

**# 40) -** An incidence of a small bat being trapped in the web of a large orb-weaving spider was witnessed on Dunk Island, Queensland (reported by Le Souef 1915; Banfield 1918; McKeown 1952). Dunk Island is a tropical rainforest island situated between Townsville and Cairns. Banfield described this incidence as follows: “…At dawn a bat flew into a spider’s web spun during the night, the extremities of the wings being so entangled that struggling was almost impossible. A big spider pounced on it. Not a minute elapsed from the entanglement until the bat was released, but the venom of the spider had done its work. There was not a sign of life…” According to Le Souef (1915), who reported the same incident (after a visit to Mr. Banfield’s property on Dunk Island), the spider species in question was *Nephila pilipes* (formerly termed *Nephila fuscipes*).

**# 41) -** McKeown (1952) reports that a vespertilionid bat was trapped in the web of an unidentified web-building spider in Urunga. Based on a photograph taken in 1944, McKeown identified the bat in question to be possibly Gould’s Wattled Bat, *Chalinolobus gouldii* (Vespertilionidae). Urunga is a small town in the Mid North Coast region of New South Wales, with a subtropical climate.

**# 42) -** In 1940, in Wamberal, New South Wales, an orb-weaving spider *Eriophora* *transmarina* (Araneidae) was observed catching two or three bats in its web which was suspended between two banana trees (McKeown 1952). Wamberal is a coastal [suburb](http://en.wikipedia.org/wiki/List_of_Central_Coast_suburbs) with a warm temperate climate. In McKeown’s report it is not mentioned whether the spider was devouring the bats.

**# 43) -** In 2009, during bat radio-tracking studies in the Cumberland State Forest, a bushland reserve near Sydney, New South Wales, one of the experimental bats got caught in the web of an orb-weaving spider (presumably a *Nephila* sp.; Threlfall 2011). The site was open forest dominated by *Eucalyptus* species and characterized by a temperate climate. The bat, an adult male Gould’s Long-eared Bat, *Nyctophilus gouldi* (Vespertilionidae), was dead on the investigator’s arrival and apparently not wrapped in silk. This may have been a case of accidental death, the bat dying from dehydration and hypothermia after crashing into the spider web (C. Threlfall, pers. comm.).

**# 44) -** An incidence of a bat being entangled in a spider web was noted at Tolmer Falls in Litchfield National Park, 100 km south of Darwin in the Northern Territory (Churchill 2008; S. Churchill, pers. comm.). The site was located in a small area of tropical rainforest, mainly Carpentaria palm trees, in a gorge, along a small waterway. There, a dead male Dusky Leaf-nosed Bat, *Hipposideros ater* (Hipposideridae) was found entangled in a *Nephila sp.* web that was constructed in close proximity to numerous other webs (Churchill 2008; S. Churchill, pers. comm.). The bat had apparently got partially tangled in one web and then fallen into the other webs before being fully caught. The bat was caught only 50 meters from a cave colony (S. Churchill, pers. comm.).

**# 45) -** At the Wau Ecological Institute, Papua New Guinea - which is surrounded by tropical forest remnants, tropical secondary forests, grasslands and coffee plantations - it was witnessed that a *Pipistrellus* bat (Vespertilionidae) became entangled in the web of a *Nephila pilipes* (Hill & Smith 1984). The spider bit the bat before it could be freed from the web; however, the bat recovered and flew away after the sticky webbing was cleaned from its wings.

**Africa**

**# 46) -** At the tropical Liwonde National Park, Malawi, a Schlieffen’s Twilight Bat, *Nycticeinops schlieffeni* (Vespertilionidae) was released during the daytime hours (Happold et al. 1987). This experiment was conducted in a landscape dominated by *Brachystegia* woodland, which is a rather open type of woodland. The bat flew into the web of a large unidentified web-building spider where it became entangled and was unable to escape. While the spider must have been alerted by the vibrations generated by the bat, it ignored it.

**# 47) -** In January 2008, Donald Schultz photographed a live insectivorous bat that had become entangled in the web of a *Nephilengys cruentata* (Nephilidae; Fig. 2F). This incidence took place at Nisela Lodge, Big Bend, Southern Swaziland, where numerous spiders had built webs in thatch roofs. *Nephilengys cruentata* is a nocturnal orb-weaving spider characterized by its synanthropic habits (Kuntner 2007). Donald Schultz (pers. comm.): “…I didn’t see the bat get tangled, but heard the noise. The spider did not immediately approach. It seemed to wait for the bat to tire. After two or three minutes, the bat decreased its movements. The spider started its approach. When the bat sensed the movement of the spider, it started struggling again and broke free…”

**# 48) -** According to Rosevear (1965), a dead Banana Pipistrelle, *Neoromicia nana* (Vespertilionidae) was found caught in the web of an unidentified web-building spider near Kenema, Sierra Leone, a place located in the African tropics.

**North America**

**# 49) -** In a lower Sonoran desert-scrub area near Yuma, Arizona, USA, the carcass of a small adult bat was found entangled in the middle of a disorganized spider web (Laduc 1993). The non-wrapped bat was identified as Western Pipistrelle, *Pipistrellus hesperus* (Vespertilionidae). It could not be determined whether the bat was fed upon because of the bat’s desiccated condition.

**# 50) -** Hermanson & Wilkins (1986) conducted a study on the mortality of neonatal bats in a maternity roost in Williston, Levy Co, Florida. The bats (Southeastern Myotis, *Myotis austroriparius*; Vespertilionidae) occupied an attic of an old, poorly maintained two story wooden frame house with a tin roof. Neonatal and juvenile *Myotis* *austroriparius* carcasses were found entangled in spider webs along the eaves.

**Europe**

**# 51) -** In May 2011, Thomas Mainitz found a dead pipistrelle bat (Vespertilionidae) entangled in the web of an ecribellate orb-weaving spider on a building site in Murrhardt, 40 km northeast of Stuttgart, Germany. Murrhardt is located in the northern temperate zone. A short report (including two photographs) of this incident was published on 27 May 2011 in the internet edition of the German tabloid *BILD* (Axel Springer newspaper publisher). The spider is not seen in the photograph and its taxonomic identity could therefore not be determined. Based on web structure and knowledge of the local spider fauna, however, it is obvious that the web builder was a species in the family Araneidae (S. Zschokke, pers. comm.).

**# 52) -** Another incidence of a bat caught in a spider web was seen by several witnesses on the Isle of Wight, South East England (G. Street, pers. comm.). This incidence occurred in 2007 at the Swiss Cottage, a known maternity pipistrelle bat roost that, on average, houses around 70 bats each year. A live adult pipistrelle (apparently Common Pipistrelle, *Pipistrellus pipistrellus*; Vespertilionidae) was found entangled in the web of an unidentified spider species in the eaves of the cottage. The bat had apparently been trapped for a couple of days and was underweight and dehydrated. After the bat was carefully freed from the web, it died on the way to the Isle of Wight Bat Hospital in Sandown (operated by Graham & Donna Street).

With regard to the two European incidences it should be noted that spiders producing giant orb-webs (1-2 m diameter), as known from the tropical and subtropical regions, are not found in Europe. The largest orb-webs known from Central Europe are those of the nocturnal araneid *Nuctenea umbratica* which constructs webs with up to 0.7 m diameter outside of buildings (Bellmann 1997). The two European incidences might have been cases of non-predation death with no involvement of the spider.

**REFERENCES**

Banfield EJ (1918) Tropic Days. London: T. Fisher Unwin Ltd. 313 p.

Bellmann H (1997) Kosmos-Atlas Spinnentiere Europas. Stuttgart: Kosmos-Verlag. 304 p.

Bhattacharya GC (1941) *Heteropoda venatoria* preying on a pipistrelle bat. Curr Sci 10: 183.

Bleckmann H, Lotz T (1987) The vertebrate-catching behaviour of the fishing spider *Dolomedes triton* (Araneae, Pisauridae). Anim Behav 35: 641-651.

Cantor T (1842) General Features of Chusan, with remarks on the Flora and Fauna of that Island. Animals Observed at Chusan. Ann Mag Nat Hist 9: 481-493

Churchill S (2008) Australian Bats, 2^nd^ edition. Sydney: New Holland. 255 p.

Conniff R (1996) Tarantulas: earth tigers and bird spiders. National Geographic 190: 99-115.

Das KSA, Sreekala LK, Abdurahiman O (2012) Predation on the Kelaart’s Pipistrelle Bat *Pipistrellus ceylonicus* Keelart (Chiroptera: Vespertilionidae), by the Reddish Parachute Tarantula, *Poecilotheria rufilata* Pockock (Araneae: Theraphosidae) in Chinnar Wildlife Sanctuary, Kerala, India. Tropical Natural History 12: 257-260.

Happold DCD, Happold M, Hill JE (1987) The bats of Malawi. Mammalia 51: 337-414.

Hermanson JW, Wilkins KT (1986) Pre-weaning mortality in a Florida maternity roost of *Myotis austroriparius* and *Tadarida brasiliensis*. J Mammal 67: 751-754.

Hill DS, Hore PM, Thornton IWB (1982) Insects of Hong Kong. Hong Kong: Hong Kong University Press. 540 p.

Hill JE, Smith JD (1984) Bats: A Natural History. Austin: University of Texas Press. 243 p.

Kaston BJ (1965) Some little known aspects of spider behavior. Am Mid Nat 73: 336-356.

Kershaw JCW (1905) II. Butterfly-destroyers in Southern China. T Roy Ent Soc London 53: 5-8.

Koh JKH (2000) A Guide to Common Singapore Spiders. Singapore: Singapore Science Centre. 160 p.

Kuntner M (2007) A monograph of *Nephilengys*, the pantropical ‘hermit spiders’ (Araneae, Nephilidae, Nephilinae). Syst Entomol 32: 95-135.

Laduc TJ (1993) Accidental death by web entanglement in the Western Pipistrelle, *Pipistrellus hesperus*. Bat Research News 34: 58-59.

Le Souef D (1915) North Queensland birds. Emu 14: 163-166.

Levi HW (1970) The *Ravilla* group of the orb weaver genus *Eriophora* in North America (Araneae: Araneidae). Psyche 77: 280-302.

Liat LB (1964) The bird-eating spider. Malay Nat J 18: 20-25.

McKeown KC (1952) Australian Spiders. Sydney: Angus and Robertson. 274 p.

Medway L (1972) Reproductive cycles of the flat-headed bats *Tylonycteris pachypus* and *T. robustula* (Chiroptera: Vespertilioninae) in a humid equatorial environment. Zool J Linn Soc 51: 33-61.

Ochoa JG, Soriano PJ, Hernandez-Camacho J (1994) Sobre la presencia de *Cyttarops alecto* (Chiroptera: Emballonuridae) en Colombia. Trianea 5: 411-414.

Platnick NI (2012) The world spider catalog, v12.5. American Museum of Natural History. Available: http://research.amnh.org/iz/spiders/catalog. DOI:10.5531/db.iz.0001.

Plumpton DL, Jones JK (1992) *Rhynchonycteris naso*. Mammalian Species 413 : 1-5.

Robinson MH, Robinson B (1976) The ecology and behavior of *Nephila maculata*: A suppIement. Smith Cont Zool 218: 1-22.

Robinson MH, Robinson B, Graney W (1971) The predatory behavior of the nocturnal orb web spider *Eriophora fuliginea* (C.L. Koch) (Araneae: Araneidae). Rev Per Entomol 14: 304-315.

Rosevear DR (1965) The Bats of West Africa. London: Publications British Museum (Natural History). 418 p.

Sherriffs WR (1934) Hong Kong spiders. I. Hong-Kong Naturalist 5: 85-90.

Suter RB, Gruenwald J (2000) Spider size and locomotion on the water surface (Araneae, Pisauridae). J Arachnol 28: 300-308.

Threlfall C (2011) Conserving biodiversity in urban landscapes. Mechanisms influencing the distribution, community assembly and resource use of insectivorous bats in Sydney, Australia. Ph.D. Thesis, Evolution & Ecology Research Centre, School of Biological, Earth and Environmental Sciences, University of New South Wales, Sydney, Australia.

Timm RM, Losilla M (2007) Orb-weaving spider, *Argiope savignyi* (Araneidae), Predation on the proboscis bat *Rhynchonycteris naso* (Emballonuridae). Caribb J Sci 43: 282-284.

Wilson DE (1971) Ecology of *Myotis nigricans* (Mammalia: Chiroptera) on Barro Colorado Island, Panama Canal Zone. J Zool 163: 1-13.

World Association of Zoos and Aquariums (WAZA) (2011) Brazilian salmon pink (*Lasiodora parahybana*). Available: <http://www.waza.org/en/zoo/visit-the-zoo/invertebrates-house/spiders-and-scorpions-1254385524/lasiodora-parahybana>. Accessed January 2011.

Young AM (1972) An experimental study on the relation of mortality of young to adult numbers in colonies of the lesser sac-winged bat, *Saccopteryx bilineata perspicillifer*. Am Mid Nat 87: 158-164.
